# Transcriptome profiling by combined machine learning and statistical R analysis identifies TMEM236 as a potential novel diagnostic biomarker for colorectal cancer

**DOI:** 10.1038/s41598-021-92692-0

**Published:** 2021-07-12

**Authors:** Neha Shree Maurya, Sandeep Kushwaha, Aakash Chawade, Ashutosh Mani

**Affiliations:** 1 Department of Biotechnology, Motilal Nehru National Institute of Technology Allahabad, Prayagraj, 211004 India; 2 National Institute of Animal Biotechnology, Hyderabad, 500032 India; 3 Department of Plant Breeding, Swedish University of Agricultural Sciences, 230 53 Alnarp, Sweden

**Keywords:** Genome informatics, Machine learning, Cancer genomics

## Abstract

Colorectal cancer (CRC) is a common cause of cancer-related deaths worldwide. The CRC mRNA gene expression dataset containing 644 CRC tumor and 51 normal samples from the cancer genome atlas (TCGA) was pre-processed to identify the significant differentially expressed genes (DEGs). Feature selection techniques Least absolute shrinkage and selection operator (LASSO) and Relief were used along with class balancing for obtaining features (genes) of high importance. The classification of the CRC dataset was done by ML algorithms namely, random forest (RF), K-nearest neighbour (KNN), and artificial neural networks (ANN). The significant DEGs were 2933, having 1832 upregulated and 1101 downregulated genes. The CRC gene expression dataset had 23,186 features. LASSO had performed better than Relief for classifying tumor and normal samples through ML algorithms namely RF, KNN, and ANN with an accuracy of 100%, while Relief had given 79.5%, 85.05%, and 100% respectively. Common features between LASSO and DEGs were 38, from them only 5 common genes namely, VSTM2A, NR5A2, TMEM236, GDLN, and ETFDH had shown statistically significant survival analysis. Functional review and analysis of the selected genes helped in downsizing the 5 genes to 2, which are VSTM2A and TMEM236. Differential expression of TMEM236 was statistically significant and was markedly reduced in the dataset which solicits appreciation for assessment as a novel biomarker for CRC diagnosis.

## Introduction

Colorectal Cancer (CRC) is very common in many countries and is one of the major causes of death worldwide^1^. According to the American Cancer Society incidence rate of CRC will increase by more than ten percent in 2020. Although improvement in screening techniques along with better treatment options have reduced the mortality rate, still the deaths from CRC have increased among people of age below 55 by 2% every year during 2007–2016^2^. CRC may be sporadic and heterogeneous. Molecular studies have found different biological pathways involved in CRC progression^3^. Despite the unprecedented growth of gene expression data in recent years, the molecular diagnosis of CRC remains a challenge and there is a strong need of finding diagnostic biomarkers having specificity and accuracy.

Technical advancement in RNA sequencing technology along with the availability of abundant public data from and The Cancer Genome Atlas (TCGA) and Gene Expression Omnibus (GEO) have accelerated the study of gene expression patterns in CRC over time. For example, Sun et al.^1^ used GEO datasets and applied the Robust Rank Aggregation method to identify significant Differentially Expressed Genes (DEGs). They found 494 significant differential expressions containing 282 downregulated and 212 upregulated genes. Enrichment analysis performed by DAVID and KOBAS found DEGs to be involved in different cancer-related functions and pathways. Another study by Su et al.^4^ has used both miRNA and mRNA datasets from GEO to identify significant genes. Total 465 overlapped DEGs from 2 datasets and 44 DEMs (Differential expressed miRNAs) were obtained. 137 targets of the DEMs were identified from the overlapped 465 DEGs. A regulatory network of the miRNA-mRNA overlapping genes was constructed and further analyzed for their roles in the regulation of cell proliferation and metastasis.

The studies which are mentioned above had only used the traditional approaches of R bioconductor for finding the genes responsible in CRC progression.

The gene expression data contains huge dimensionality, so the feature selection methods are adopted accordingly^5^. Sometimes the traditional approaches often provide results that are inconsistent in behavior. In this context, alternative methods can be implemented which can provide better and consistent results to achieve the respective goal. The classification of gene expression data can be performed through machine learning (ML) algorithms to find significant features.

Studies conducted by Wang and Gotoh, had used canonical a depended degree-based feature selection approach for gene selection from microarray datasets of colon, breast, lung, prostate, leukemia and central nervous system^6^. Liu et al., had implemented Robust principal component analysis (RPCA) for colon cancer dataset to identify the differentially expressed genes between the tumor and normal tissues^7^. Loscalzo et al., had implemented consensus group stable (CGS) feature selection method for colon, leukemia, lung, and prostate cancer datasets to identify key features involved in the progression of the respective cancer^8^.

The CRC dataset in th present study was compared by using two feature selection methods; Least Absolute Shrinkage and Selection Operator (LASSO)^9^ and Relief^10^ with three classifying algorithms, Random Forest^11^, K-Nearest Neighbour (KNN)^12^, and Artificial Neural Network (ANN)^13^.

Since the studies which were conducted had either used statistical approach or ML based approach only for the genes identification but the combined hybrid approach was never used in practice. Our aim of the present study is to propose a simple and unique approach to find significant features from the gene expression dataset of CRC by assessing the ability of different ML algorithms along with the power of statistical significance of R bioconductor packages.

## Results

### Dataset overview

**Figure 1:**
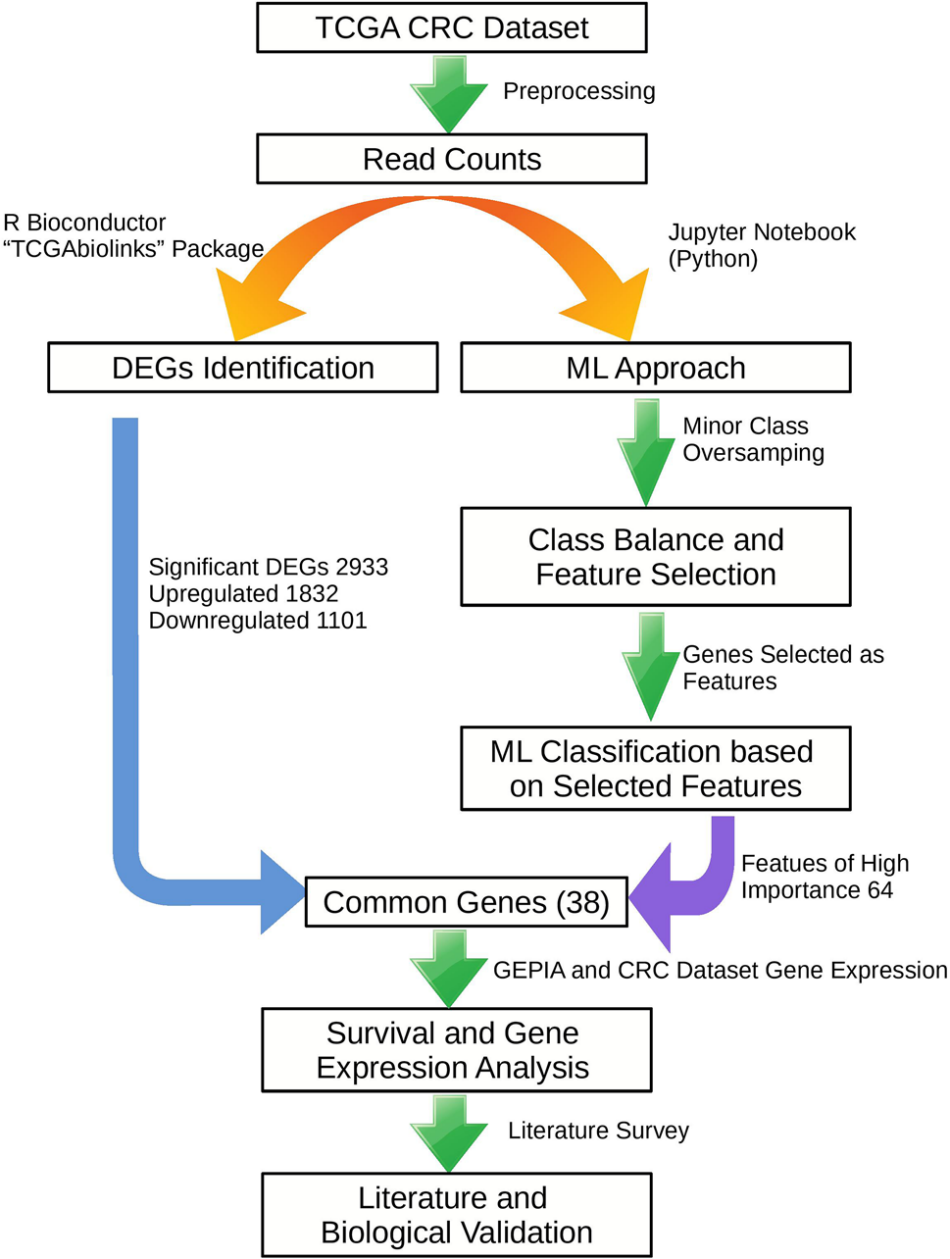
Workflow for the identification of novel biomarkers for colorectal cancer. TCGA: The Cancer Genome Atlas; CRC: colorectal cancer; DEGs: differentially expressed genes; ML: machine learning; GEPIA: gene expression profiling interactive analysis.

**Figure 2:**
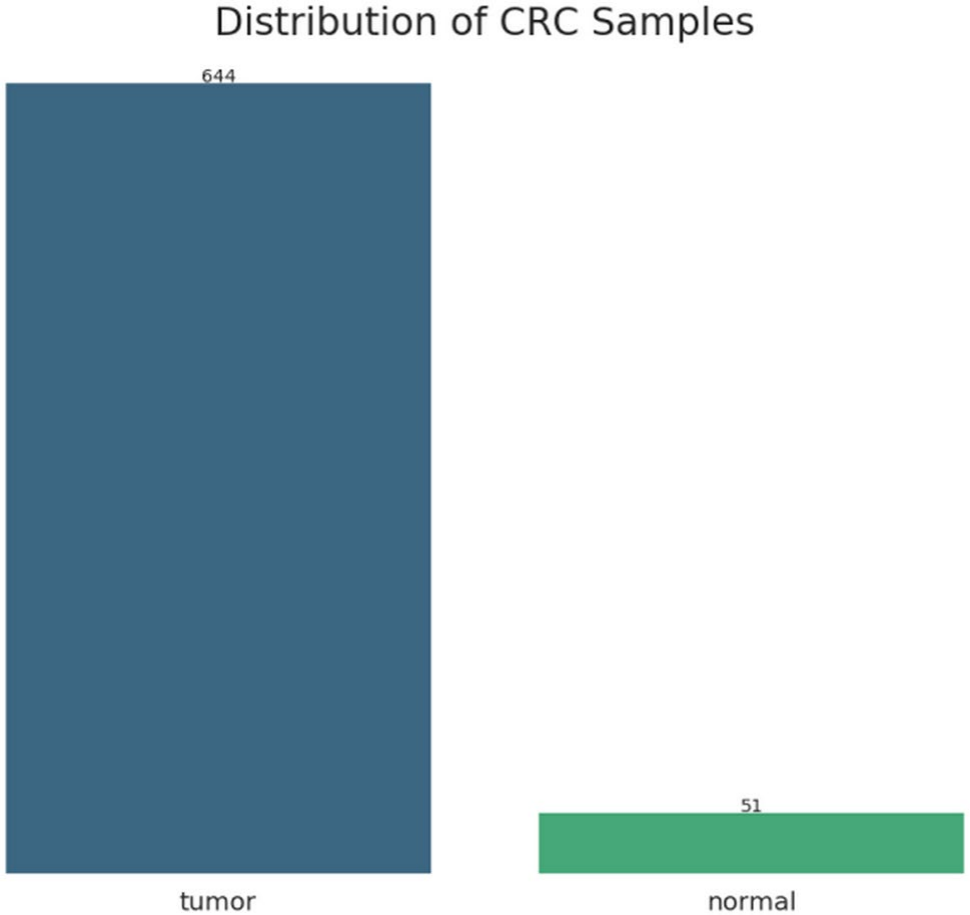
Distribution of CRC tumor and normal tissue samples in the working dataset. Blue color shows the number of CRC samples, green colour shows the normal tissue samples.

In the present study, the TCGA CRC dataset was analyzed by combining the statistical and ML approach Fig. 1. A total of 695 CRC samples were collected from the TCGA database Fig. 2. The thresholds for obtaining the normalized mRNA data includes data type of Gene Expression Quantification, workflow type of HTSeq-Counts with correlation cut-off of 0.6 in Supplementary data file S1.

**Figure 3:**
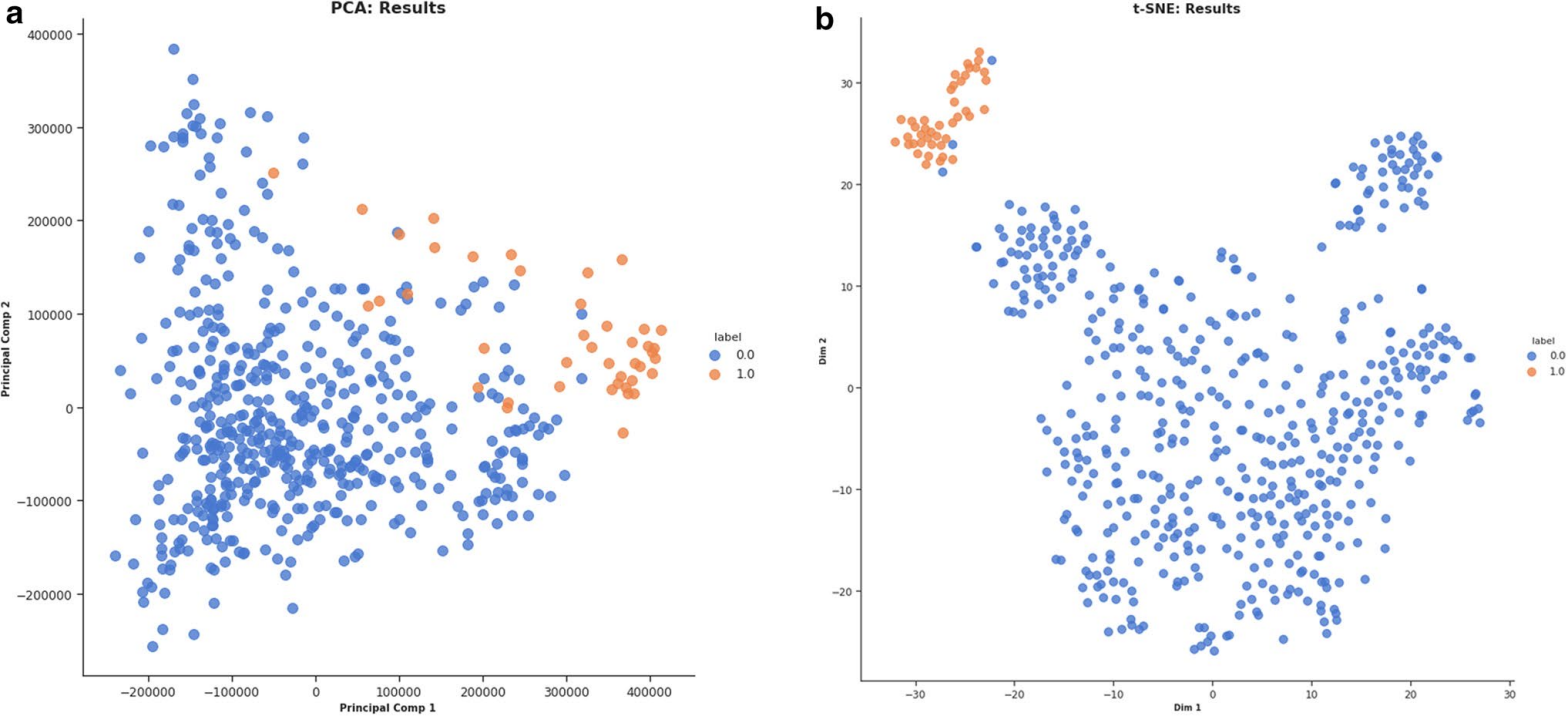
Dimensionality reduction analysis for CRC dataset. (**a**) PCA analysis. (**b**) t-SNE analysis. 0 stands for CRC tissue samples while 1 stands for normal tissue samples.

The CRC gene expression dataset was reduced in dimensionality and was further analyzed through the different algorithms, named as Principal Component Analysis (PCA) and t-distributed stochastic neighborhood estimation (t-SNE). The performance of the t-SNE algorithm was found to have better accuracy than PCA in classifying the samples Fig. 3.

### Identification of differentially expressed genes

**Figure 4:**
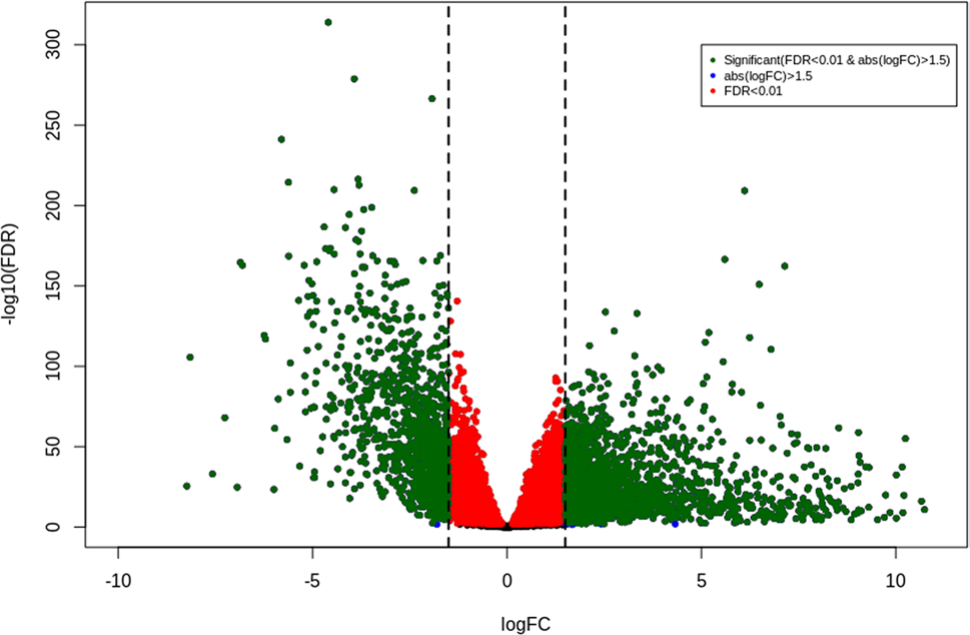
The volcano plot of the distribution of DEGs in CRC dataset. Green—significant DEGs(|logFC > 1.5| && FDR 0.01), red—FDR > 0.01 and blue—|logFC > 1.5|.

After the comparative analysis of the CRC and normal tissue samples 2933 DEGs were obtained. I included 1832 upregulated and 1101 downregulated, as shown by the volcano plot for the CRC dataset in Fig. 4 and Supplementary data file S2.

### Class imbalance and feature selection

**Figure 5:**
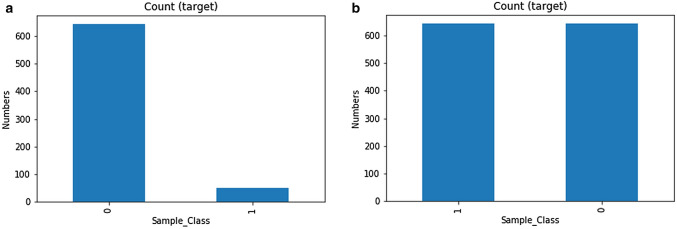
CRC Dataset classes. (**a**) dataset before balancing the classes, and (**b**) dataset after oversampling to achieve class balance. 0—Tumor, 1—Normal.

The obtained dataset was highly imbalanced with the normal class having 51 and tumor class had 644 samples. This kind of data can produce biased results while doing the analysis having the of tumor to normal sample ratio of 12.63: 1 while the normal samples containing only 7.9 percent of the total sample space, as shown in Fig. 5 and Supplementary data file S3.

So, before applying the feature selection methods class balancing was performed using the oversampling technique. This resulted in the equal distribution of normal and tumor samples. LASSO (Regularization based embedded method) had provided 64 features with 5000 iterations and alpha of 0.001 while, Relief is a filter-based feature selection method which provides the feature scores for the dataset (high score with high importance).

### Machine learning analysis

**Figure 6:**
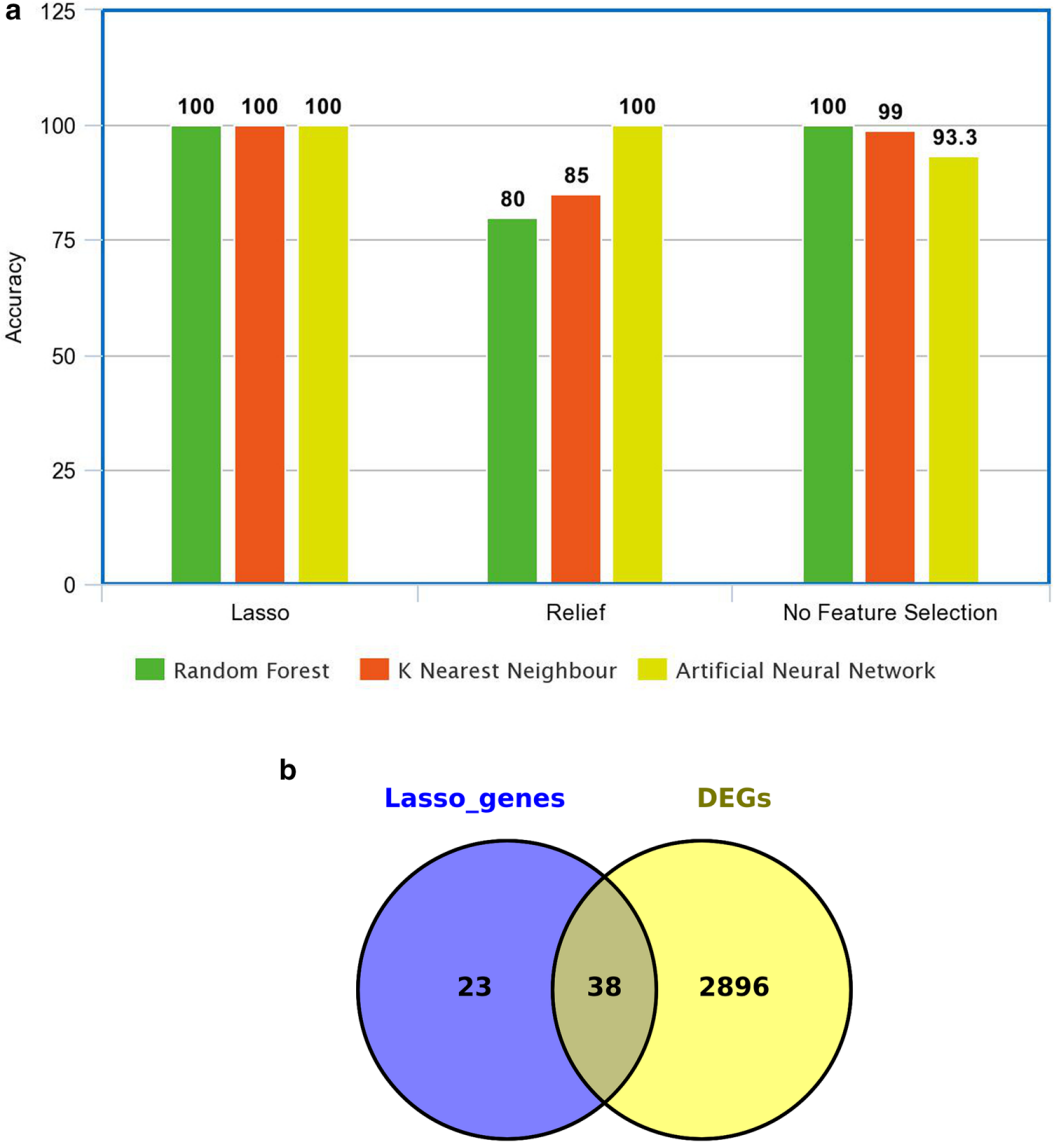
ML analysis of the CRC dataset. (**a**) Accuracy of the selected ML algorithms. (**b**) Venn diagram of a common set of overlapping genes obtained from ML approach and DEGs.

CRC dataset was classified initially without balancing the tumor and normal class to assess the performance of selected ML algorithms while classifying the data. As previous literature suggests that RF algorithm outperforms the ANN and KNN both with the accuracy of 100%. Although the classes were not balanced and the obtained results could be biased so, the dataset was balanced and feature selection methods were applied to extract the best set of features for classification. After feature selection again ML algorithms were applied to check the accuracy of the models. LASSO has given the best results in terms of accuracy as compared to the Relief feature selection method for classification as shown in Fig. 6a.

Based on accuracy LASSO had performed better in classifying the CRC dataset. So, features obtained from LASSO were found to be overlapping with DEGs obtained from the Bioconductor R package (TCGAbiolinks), and the common DEGs were selected for further analysis, as shown in Fig. 6b by Venn diagram^14^.

### Biomarker gene selection based on gene expression and survival analysis

**Figure 7:**
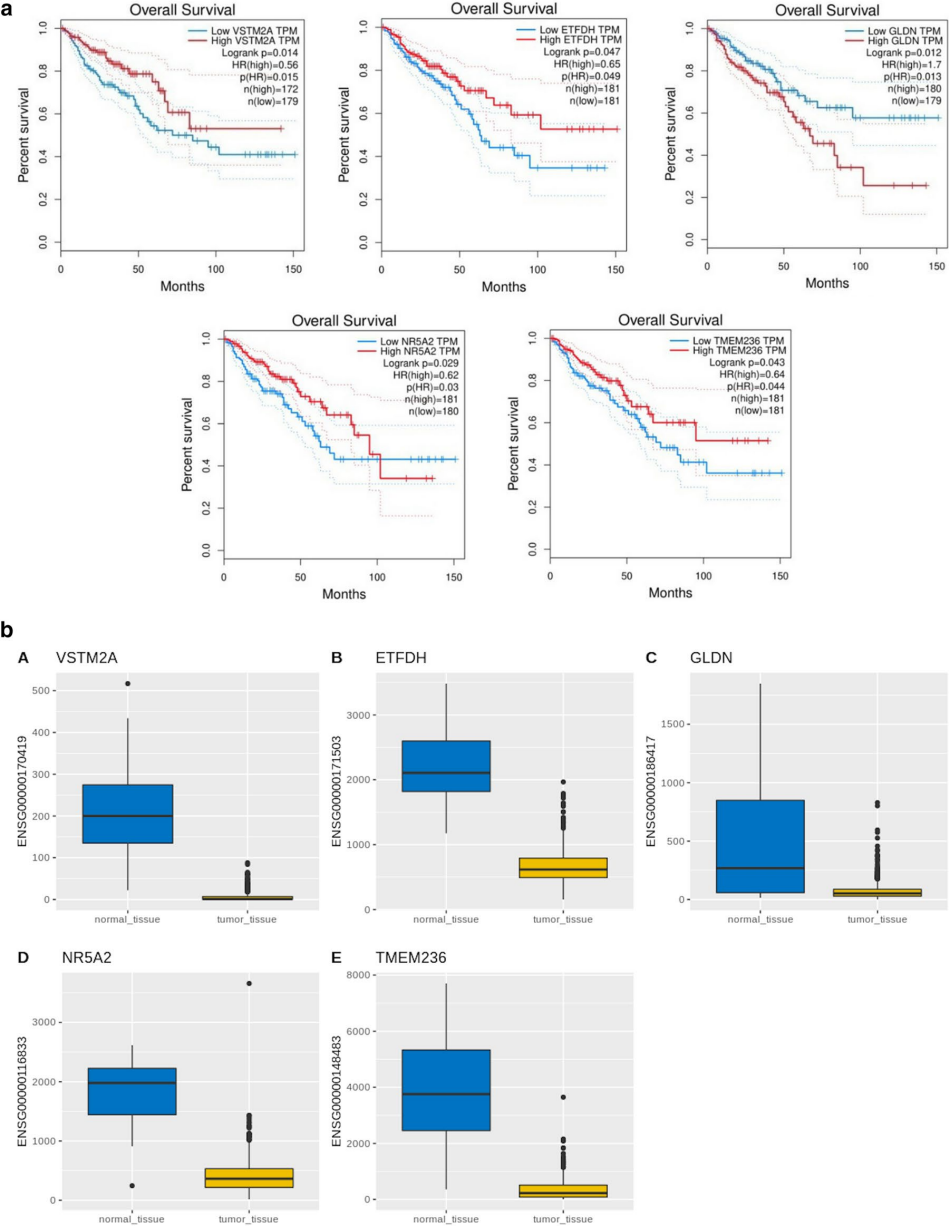
Survival and gene expression analysis. (**a**) Kaplan–Meier survival analysis of VSTM2A, ETFDH, GLDN, NR5A2, and TMEM236 for CRC dataset. (**b**) Gene expression of the VSTM2A, ETFDH, GLDN, NR5A2, and TMEM236 for CRC dataset from TCGA database. Blue: Normal tissue and Yellow: CRC tumor tissue.

Total 38 genes were selected for further analysis. Their gene expression profiles were and regulatory features were analyzed in CRC samples and were compared to normal samples. The shortlisted 38 genes were further filtered through GEPIA online database based on the overall survival analysis. Total 5 genes namely VSTM2A (log-rank *p* = 0.014), ETFDH (log-rank *p* = 0.047), GLDN(log-rank *p* = 0.012), NR5A2(log-rank *p* = 0.029), and TMEM236(log-rank *p* = 0.043) were significantly correlated with the overall survival of the CRC patients as shown in Fig. 7a. Gene expression of the finally shortlisted genes was explored for the CRC dataset as shown in Fig. 7b. The function of finally enlisted genes is summarized in Table 1.

**Table 1:** List of genes significant for classifying CRC dataset based on the literature.

S. no.	Gene name	Function
1	ETFDH	Component of the electron-transfer system in mitochondria and accepts electrons from ETF and reduces ubiquinone
2	NR5A2	Nuclear receptor that acts as a key metabolic sensor by regulating the expression of genes involved in bile acid synthesis, cholesterol homeostasis, and triglyceride synthesis
3	TMEM236	Protein that spans the entire width of the lipid bilayer and to which it is permanently anchored. Many TMEMs functions as channels to permit the transport of specific substances across the biological membrane^15^
4	VSTM2A	It has a role in the regulation of the early stage of white and brown preadipocyte cell differentiation^16^
5	GLDN	Promotes formation of the nodes of Ranvier in the peripheral nervous system

### Selection of biomarker genes for CRC

**Figure 8:**
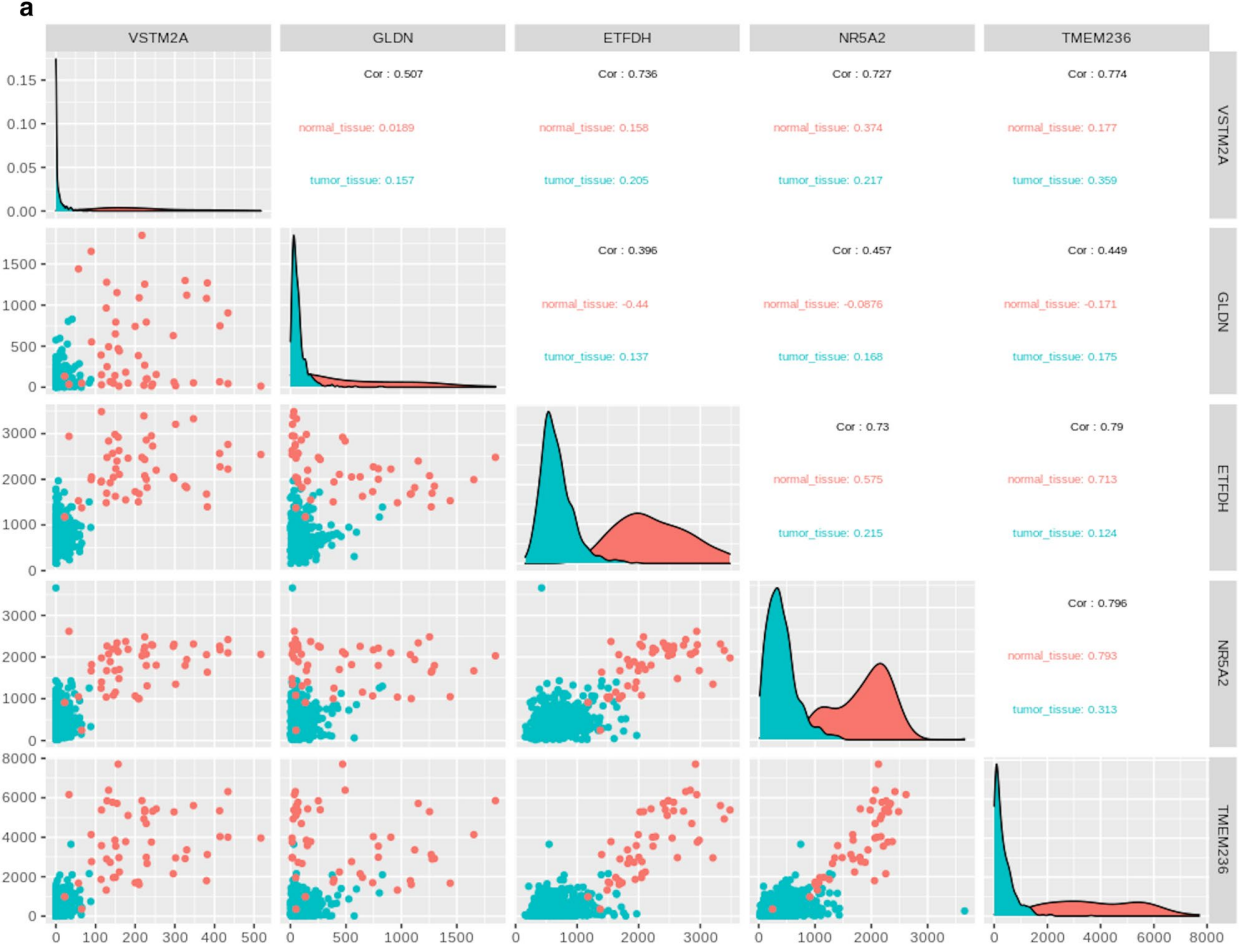

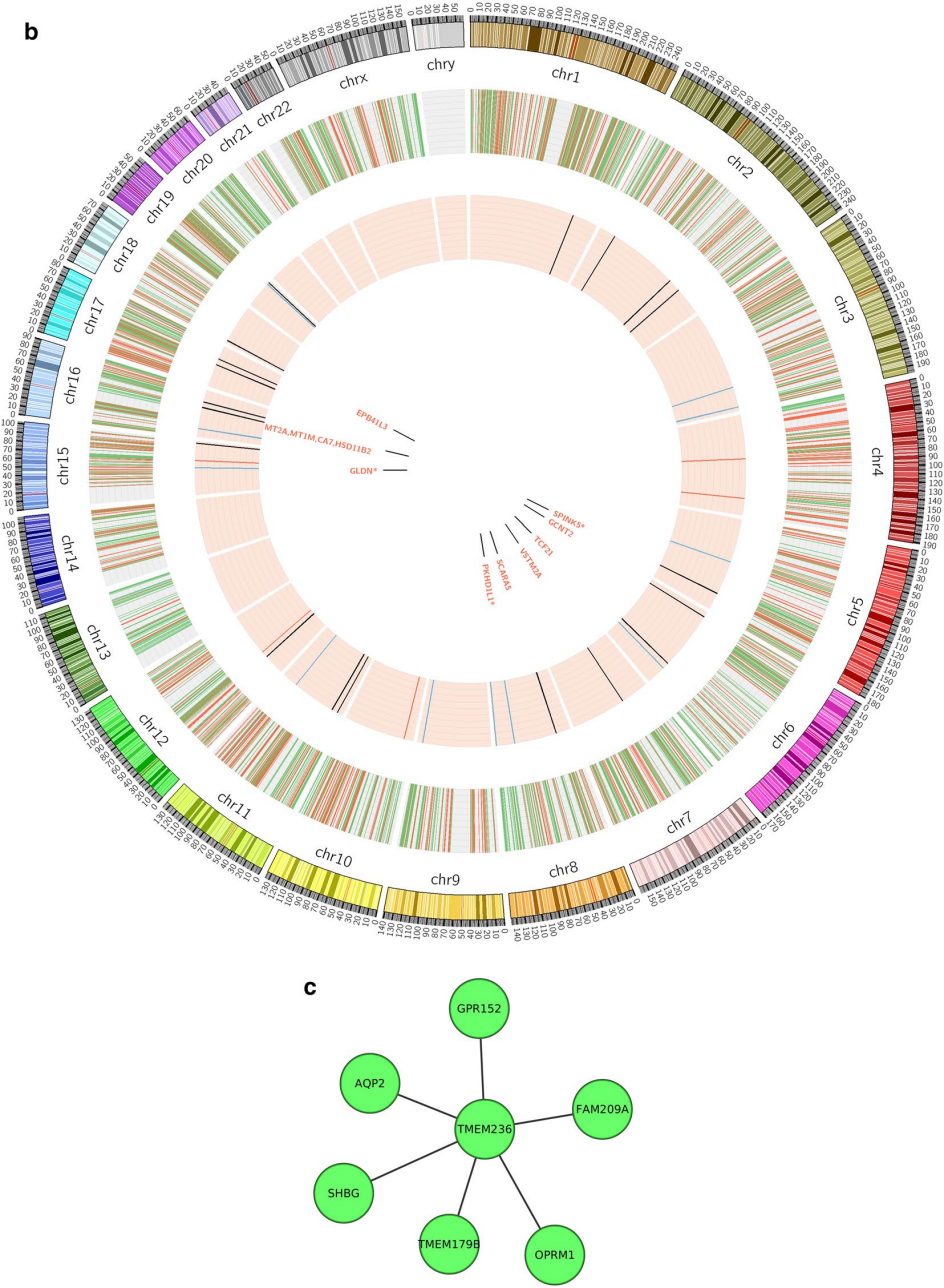
Correlation analysis and location of significant genes. (**a**) Correlation plot between final selected genes (VSTM2A, GLDN, ETFDH, NR5A2, and TMEM236), (**b**) Circos plot to show the genomic location of the selected genes where the outermost band shows the karyotype and the circle inside that shows the total DEGs obtained through traditional approach while the circle inside that with pink background shows the final selected 38 common genes between ML and traditional DEG approach. Red color: Normal tissue samples, and Blue color: CRC tumor tissue samples, and (**c**) gene interaction pathway for TMEM236 gene.

The 5 selected genes namely, VSTM2A, NR5A2, TMEM236, GDLN, and ETFDH we considered as potential biomarkers. Correlation among the selected 5 genes is shown in Fig. 8a and the location of the selected genes in the human genome is shown through the circos plot in Fig. 8b. On the basis of pathway analysis two genes (TMEM236 and VSTM2A) were found promising. however TMEM236 downregulation is more statistically significant in CRC data. The gene interaction pathway for the TMEM236 gene is shown in Fig. 8c. TMEM are transmembrane proteins that span biological membranes. Although till now the function of TMEM236 is unknown and its relevance to CRC is still need to be explored. But experimental pieces of evidence suggest that TMEM proteins can be described as tumor suppressors or oncogenes^15^. TMEM236 is significantly downregulated as found in the CRC expression dataset which may be indicative of its property. VSTM2A downregulation is also known to be associated with poor survival of CRC patients and antagonist of canonical Wnt signaling by directly binding to LRP6 and inducing LRP6 endocytosis and degradation^16^.

## Discussion

In the present study, an integrated ML and bioinformatics analysis approach was combined to identify diagnostic biomarker genes for CRC. Based on the CRC gene expression count data 695 samples were obtained out of which 644 were tumor samples while 51 were normal samples. Initially ML approach was implemented on the CRC dataset with and without class balancing. The ML algorithms namely, RF, KNN, and ANN, which were used initially without feature selection technique and class balancing. This shows that RF had outperformed in classifying the samples of tumor and normal classes as compared to the remaining algorithms.

Since the normal and tumor classes have a huge imbalance the classification could have been the result of class biases. Later on feature selection techniques, LASSO and Relief were implemented on the CRC dataset along with ML algorithms. The best results obtained from feature selection with ML were overlapped with those obtained from the traditional approach of obtaining DEGs (using R Bioconductor package). Accuracy evaluation was achieved with Leave-One-Out-Cross-Validation (LOOCV)^17^ method.

Our findings demonstrate the negative effect of non-informative genes on the classification and feature selection. It was found that the performance of the ML algorithms was better with genes selected after the feature selection methods. The total number of features were 23,186 which were reduced down further by using filter-based relief and regularization based embedded LASSO methods.

LASSO with RF had given the best results for the CRC dataset classification into a tumor and normal class with an accuracy of 100%. LASSO identified 64 genes as features for the CRC dataset classification. Total 2933 DEGs were identified through Bioconductor R package TCGAbiolinks, including 1832 upregulated and 1101 downregulated genes. Both the set of genes were overlapped and 38 common features were obtained. The selected 38 genes were further analyzed by performing their survival analysis through GEPIA and out of those 5 genes were further selected based on their log-rank *p*-value ≥ 0.05. The selected genes were VSTM2A (log-rank *p* = 0.014), ETFDH (log-rank *p* = 0.047), GLDN(log-rank *p* = 0.012), NR5A2(log-rank *p* = 0.029), and TMEM236(log-rank *p* = 0.043) and their gene expression were analyzed from the dataset.

In the previous study by Sun et al.^4^, have identified the DEGs from the GEO datasets using the robust rank aggregation method which is a statistical method and ranks the genes based on their significance score and keeps the statistically significant ones for further study. Their study had not explored the feature selection aspect with ML-based analysis to confirm their obtained genes list rather they had gone for survival analysis through GEPIA. But the present study had gone for both the statistical and ML-based approach to confirm the involvement of selected genes in the CRC progression.

Functional review and analysis of the selected genes helped in downsizing the 5 genes to 2, which are VSTM2A and TMEM236. VSTM2A has a role in the regulation of preadipocyte cell differentiation. It was found that VSTM2A gene expression was markedly reduced in the CRC dataset tumor samples in comparison to normal samples. Studies have shown that downregulation of VSTM2A protein and VSTM2A DNA promoter hypermethylation is associated with poor survival of CRC patients and hyperactivation of the Wnt/β-catenin signaling pathway is a critical step in colorectal tumorigenesis. The interaction of VSTM2A with LRP6 initiates an intracellular signal responsible for Wnt inhibition. It happens by inhibition of LRP6 phosphorylation and suppression of LRP6 protein expression which is induced by VSTM2A protein availability in a dose-dependent fashion^18^. The receptor endocytosis often occurs as a result of ligand binding with its receptor^19^. Studies have suggested that VSTM2A induces endocytosis and lysosome-mediated degradation of VSTM2A protein.

TMEM is transmembrane proteins that span the lipid bilayer and remains permanently anchored to it. TMEM is known to express differentially in many cancers such as in hepatic cancer (TMEM7)^20^, lymphomas (TMEM176)^21^, and colorectal cancer (TMEM25)^22^. TMEM236 differentially expresses itself in the CRC dataset where its expression is downregulated in tumor samples as compared to normal samples. The gene interaction network pathway analysis shows TMEM236 interaction with TMEM179B, OPRM1, FAM209A, GPR152, AQP2, and SHBG genes in Supplementary Data File S4. Out of these 6 interactors OPRM1, AQP2, and SHBG have available studies suggesting their role in CRC progression. The μ-Opioid receptor gene (OPRM1) is an important element in cancer opioid analgesic effectiveness^23^. Preclinical evidence has shown increased expression of the OPRM1 gene in patients with CRC but there is no association with mortality or increased risk of reoccurrence^24^ and its role in cancer stage and genetic polymorphism has to be studied further. The aquaporin2 (AQP2) gene is important for controlling water permeability in cells. AQPs expression is may be involved in the development of human cancer due to its serum-responsive nature^25^. Reports suggest that high expression of AQPs in tumor cells have an association with an early stage CRC development and its expression study can lead to a better understanding of colorectal carcinogenesis. The sex hormone binding-globulin gene (SHBG) is hepatically derived and transporter of sex hormone have a positive relationship with CRC risk in men^26^ and has an inverse association with the ratio of estradiol to testosterone and CRC in postmenopausal women^27^. As women are at lower risk than men for CRC, a study conducted by Mori et. al., h found that circulating testosterone levels from blood analysis has shown a positive association with CRC progression risk^28^.

The difference in the expression level between normal and tumor samples is huge for TMEM236. As in previous studies, TMEM25 shows downregulation in tumor samples as compared to the normal samples and has been proven to act as a tumor suppressor in CRC. The same kind of expression pattern is found for TMEM236 and this gene could be further studied to find its role in CRC prediction study as a novel biomarker.

## Conclusion

Despite significant advancements in cancer studies early diagnosis of CRC remains a challenge. This study apprehends the differential expression of genes in 644 samples to identify novel biomarkers for CRC. Combined machine learning approaches suggested that expression levels of TMEM proteins, which are transmembrane proteins that span the lipid bilayer and remain permanently anchored to it, significant changes in CRC. Most importantly, TMEM236 is a novel gene that is significantly downregulated in colorectal tumors. However, no studies are available for TMEM 236 and their correlation with cancer, especially with colorectal cancer. The gene expression analysis suggests that TMEM236 could serve as a novel biomarker for the diagnosis of CRC.

## Methods

### CRC dataset collection and preprocessing

The CRC mRNA expression dataset was downloaded from NIH-GDC (Genomic Data Commons DataPortal) https://portal.gdc.cancer.gov-/ through Bioconductor R package TCGAbiolinks^29^. The mRNA dataset contained 695 samples, including 644 CRC tissue samples and 51 normal tissue samples.

### Identification of differentially expressed genes

CRC gene expression data was corrected, filtered, and normalized using the TCGAbiolinks package in R. Genes were filtered by setting a threshold value of 0.30 with qnt.cut (threshold selected as mean for filtering) to filter the genes with less mean score parameter. DEGs in CRC tissue samples were compared with the control samples and were screened using the edgeR by glmLRT (fit a negative binomial generalized log-linear model to the read counts for each gene) method with the FDR cut-off of 0.01 and |log 2‐FC|> 1.5.

### Class imbalance and feature selection

For enhanced classification performance of our model, we solved the class imbalance problem by applying the re-sampling technique. Re-sampling of the data can be performed in two ways (a) adding data to the minority class, also known as over-sampling, (b) deleting some of the data from the majority class, known as under-sampling. Oversampling of the data was preferred over under-sampling to minimize information loss.

The CRC gene expression dataset contained 23,186 features (genes) for 695 samples. Presence of. LASSO and Relief feature selection algorithms were used for narrowing down the number of genes as a high number of features with the dataset makes it difficult to classify the data. The Least Absolute Shrinkage and Selection Operator (LASSO) algorithm constructs a linear model and penalizes the regression coefficients with L1 distance. Most coefficients are reduced to zero and the remaining inputs are selected. Relief algorithm calculates feature value differences with nearest-neighbor pairs. Later, these feature scores are used as important values for the system. A high feature score is correlated with greater significance.

### Machine Learning analysis of gene expression data

Classification is one of the important aspects of machine learning. We here implemented three widely used ML algorithms Random Forest, KNN, and ANN for classification.

#### Without feature selection and class balancing

Initially, the ML algorithms were implemented on the gene expression data without balancing the normal class concerning the CRC class and the performance of different algorithms was evaluated.

#### Feature selection and class balancing

Balanced class gene expression data with selected features were analyzed by using different ML algorithms for classification. The accuracy and training time was calculated to find the best performing algorithm.

### Biomarker gene selection based on survival analysis and gene expression

The HT-seq counts data from the TCGA database for CRC were analyzed. The difference between the normal and tumor samples was recorded. The Gene Expression Profiling Interactive Analysis (GEPIA) online database (http:// gepia.cancer-pku.cn/; Tang et al., 2017) was used for survival analysis of the genes as prospective biomarkers.

### Selection and validation of biomarker genes based on literature

A literature survey was performed to validate the set of biomarker genes obtained from the above-mentioned procedure. Relevant literature has helped in biological validation for the final set of genes.

## Supplementary Information

Supplementary Data File 1.

Supplementary Data File 2.

Supplementary Data File 3.

Supplementary Data File 4.

Supplementary Information
