# Supplementary material for: Transcriptome profiling by combined machine learning and statistical R analysis identifies TMEM236 as a potential novel diagnostic biomarker for colorectal cancer
Source: Sci Rep. 2021 Jul 12;11:14304. doi: 10.1038/s41598-021-92692-0 (PMC8275802; doi:10.1038/s41598-021-92692-0)
Supplement: Supplementary file 5 — Supplementary Information [file 41598_2021_92692_MOESM5_ESM.docx]

**Supplementary Data Files**

**Supplementary Data File 1|** The quantified normalized read counts of mRNA gene expression data for 695 CRC sample dataset.

**Supplementary Data File 2|** Identified Differentially expressed genes between the CRC and normal tissue samples obtained after the analysis.

**Supplementary Data File 3|** Imbalanced CRC datatset normal class having 51 and tumor class 644 samples and distribution proportion of tumor: normal (12.63: 1) where normal samples only contains 7.9 percent of all the sample space.

**Supplementary Data File S4**| Network file for TMEM236 form IntAct server with 6 interactors (TMEM179B, OPRM1, FAM209A, GPR152, AQP2, and SHBG) on default node view of the graph.
